# Analysis of Inducible Nitric Oxide Synthase Gene Polymorphisms in Vitiligo in Han Chinese People

**DOI:** 10.1371/journal.pone.0027077

**Published:** 2011-12-21

**Authors:** Ying Zhang, Chunying Li, Kai Li, Ling Liu, Zhe Jian, Tianwen Gao

**Affiliations:** Department of Dermatology, Xijing Hospital, The Fourth Military Medical University, Xi'an, China; Charité-Universitätsmedizin Berlin, Germany

## Abstract

**Background:**

Vitiligo is a chronic depigmented skin disorder with regional melanocytes depletion. The pathogenesis was not completely clarified. Recently, more and more evidence suggested that polymorphisms of some genes are associated with vitiligo risk. Here, we want to examine the association between the inducible nitric oxide synthase (iNOS) gene polymorphisms and the risk of vitiligo in Chinese populations.

**Methods and Principal Findings:**

In a hospital-based case-control study of 749 patients with vitiligo and 763 age- and sex-matched healthy controls, three polymorphisms of *iNOS* gene were genotyped by using the PCR-restriction fragment length polymorphism (PCR-RFLP) and mutagenically separated PCR (MS-PCR) methods, respectively. We found the *iNOS*-954 polymorphism was associated with a significantly higher risk of vitiligo (adjusted OR = 1.36, 95% CI = 1.02–1.81). Furthermore, this association is more pronounced in vulgaris vitiligo, active vitiligo and vitiligo without other autoimmune diseases in the stratification study. Analysis of haplotypes showed increased risk for the *C*_-1173_ *C*_-954_ *C_Ex_*_16+14_ (OR = 1.44, 95% CI = 1.01–1.74). In addition, the serum iNOS activity is significantly associated with *iNOS*-954 combined genotype (*GC+CC*) and is much higher in vitiligo patients than in the controls (*P*<0.01). Logistic regression analysis of iNOS activity showed increased risk between higher activity and *iNOS*-954 *G*→*C* variant genotype carriers (*P_trend_*<0.001).

**Conclusions and Significance:**

*INOS* gene polymorphisms may play an important role in the genetic susceptibility to the development of vitiligo.

## Introduction

Vitiligo is an acquired depigmentary disorder characterized by the appearance of white patches resulting from the loss of functional melanocytes and melanin from the skin and mucous. It affects 0.1–2% of the world population [1] without preference for a specific gender [2]. Several theories about the pathomechanism of vitiligo have been suggested, including: autoimmune, neural, radical, self destruction and inherent defect theories [3]. However, none of them can explain the pathomechanism of vitiligo perfectly.

Nitric oxide (NO), a short-lived, free radical gas, act as an intercellular messenger in most or all mammalian organs, playing a major role in diverse physiologic processes and pathologic conditions [4], [5]. Many studies suggest that NO is involved in the inhibition of cell proliferation, differentiation, and apoptosis and, thus, may contribute to the pathogenesis of various autoimmune diseases [2], [6], [7]. NO is synthesized by a group of enzymes called NO synthase (NOS). NOS catalyses the production of NO and L-citrulline from L-arginine, O_2_ and NADPH. NOS family consists of three isoforms: neuronal NOS (nNOS), endothelial NOS (eNOS), and inducible NOS (iNOS) [8], [9], [10], [11]. ENOS and nNOS produce NO within seconds and its activities are direct and short acting, whereas iNOS produces very large, toxic amounts of NO in a sustained manner. Expression of iNOS is under the regulation of several cytokines such as interferon gamma (IFN-γ), tumor necrosis factor (TNF-α), and interleukin 1 beta (IL-1β) and its function is part of the macrophage-mediated response to infectious agents. It has reported that normal human melanocytes will express iNOS following incubation with LPS, TNF-α, and IFN-γ, then high doses of NO can result in self-destroy of cultured normal human melanocytes and pigment loss [12], [13], [14]. Moreover, NO is reported as a regulatory mediator involved in the development of autoimmune disease [15] and Ivanova *et al.* [14] also reported that NO can affect adherence of melanocyte and cell matrix, inhibiting melanocyte proliferation, changing melanocyte form and induced melanocyte death ultimately. Therefore, it is conceivable that iNOS is involved in the pathogenesis of vitiligo.

The human *iNOS* gene is located on the chromosome 17q11.2–q12, has a genomic size of 48 kb, and encodes a protein of 131 kDa [16]. Among several single nucleotide polymorphisms (SNPs) in *iNOS* that have been reported previously, the promoter polymorphisms *iNOS*-1173 *C*→*T* and -954 *G*→*C* have been shown to modify *iNOS* transcription and mRNA levels [17], [18]. A *C*-to-*T* transition at exon 16 was located only six amino acids N-terminally from the deletion reported by Daff [19], and the amino acid substitution (from serine to leucine) in exon 16 might be of functional interest.

Considering the important role that iNOS plays in the production of NO and, thus, in the pathogenesis of vitiligo, we hypothesized that the functional polymorphisms in the *iNOS* gene may be associated with the risk of vitiligo. However, to date, there has been no research about the polymorphisms of *iNOS* gene in the etiology of vitiligo. We genotyped the three reported functional single nucleotide polymorphisms of the *iNOS* gene (i.e., -1173 *C*→*T*, -954 *G*→*C*, and *Ex*16+14 *C*→*T*) to test this hypothesis in our ongoing hospital-based, case-control study on vitiligo in a Chinese population.

## Materials and Methods

### Subjects

This study included 749 Han Chinese patients with vitiligo and 763 age- and sex-matched controls. Because genotype frequencies can vary among ethnic groups, only Han Chinese patients (more than 90% of population of China) and controls were included in this analysis. All the patients had not subjected to any therapy in recent 6 months. All the healthy subjects did not show clinical evidence or family history of vitiligo or of any other autoimmune disorder. Vitiligo was clinically characterized in patients as segmental and non-segmental [20]. Segmental vitiligo was diagnosed if the disease followed a dermatomal distribution. Active vitiligo was defined as the appearance of new lesions or the enlargement of existing lesions in the 3 months before presentation. The protocols used in the study were approved by the Hospital's Protection of Human Subjects Committee (Xijing Hospital, Fourth Military Medical University, Clinical Ethics Committee). We have obtained informed consent from all participants involved in our study. This consent was written and saved in our department.

### Genotyping

Genomic DNA was extracted from cell pellets by using a blood genomic DNA purification kit (Tiangen, Beijing, China). The *iNOS*-954 *G*→*C* and *Ex*16+14 *C*→*T* polymorphisms were determined using the PCR-RFLP method, whereas the *iNOS*-1173 *C*→*T* polymorphism was carried out using MS-PCR. Genomic DNA was prepared from venous blood samples using a DNA extract kit (Tiangen, Beijing, China). DNA purity and concentration were determined by spectrophotometric measurement of absorbance at 260 and 280 nm. PCR was used to amplify the fragments that contained the select *iNOS* polymorphic sites. The primers of *iNOS*-954 *G*→*C* were as follows: 5′- CATATGTATGGGAATACTGTATTTCAG -3′ (forward) and 5′- TCTGAACTAGTCACTTGAGG -3′ (reverse), as previously reported [18]. The *Ex*16+14 *C*→*T* was amplified with following primers: 5′- CATATGTAAACCAACTTCCGTG -3′ (forward) and 5′- GGCAGGGCTAGGAGTAGGAC -3′ (reverse), as previously reported [21]. The primers of *iNOS*-1173 *C*→*T* are as follows: 5′- GACAAGAAGGAAATGAGTGGACACAGGTAGCAAAGTGTTGAGAC -3′ (MS-P2F), 5′- GCATTTTTCCATCATAAAAGTAA -3′ (MS-P3R) and 5′- GTGGTAGCAAATGTTGGAAT -3′ (MS-P4F), as previously reported [22].The amplified PCR products for the *iNOS*-954 *G*→*C* and *Ex*16+14 *C*→*T* polymorphisms were 573 bp and 219 bp, respectively. Then we used BsaI and Tsp509I restriction enzymes (New England Biolabs, Beverly, Mass) to delineate the *iNOS*-954 *G*→*C* and *Ex*16+14 *C*→*T* polymorphisms, respectively. Cuts by these enzymes resulted in 573-bp and 446-bp fragments in the case of the *iNOS*-954 *G* allele, 175-bp and 44-bp fragments in the case of the *iNOSEx*16+14 *C* allele. The amplified PCR products of the *iNOS*-1173 *C* and *iNOS*-1173 *T* alleles were 131 and 102 bp, respectively. About 10% of the samples were randomly selected and genotyped again with the same method to test the discrepancy rate, and the results were 100% concordant. In addition, every genotype was sequenced to confirm its authenticity.

### Serum iNOS activity analysis

We used computer-generated random numbers to assign 89 vitiligo patients from experimental group and 89 normal samples from control group for serum iNOS activity analysis. The statistical analysis of the epidemiologic feature and genotype frequency between the selected 89 vitiligo patients and experimental group did not show significance. The unit of serum NOS activity is defined as: one milliliter serum generates 1 nmol NO per minute is defined as one unit of activity. NOS has three major types: neuronal NOS (nNOS), endothelial NOS (eNOS) and inducible (iNOS). nNOS' and eNOS' existence is depended on the calcium and resided in the neuron and the endotheliocyte; however, iNOS resided in the macrophage and its existence has no relationship with the calcium. According to this principle, we could type the NOS easily. Here, we used the NOS examining kit (The first substation of Jiang Cheng bioengineering institute, Nanking) to detect the serum iNOS activity in differential samples, the experimental process was performed according to the instruction manual and previously reported [23]. Briefly, under the 530 nm wavelength, we first detected the absorbance of differential samples with the chromatometry method, then according the following formula to calculate the iNOS activity. The calculating formula is: iNOS (U/mL) = (iNOS measuring tube OD value – blank tube OD value)/coloration material nanomole absorbancy index×(bulk volume of the reaction liquid/sampling volume)×(1/optical path of the shade selection×reation time)/1 000.

### Statistical analysis

The chi-square test was used to evaluate the differences in frequency distribution for the selected demographic variables, including each allele and genotype of the *iNOS* polymorphisms and the serum iNOS activity between the vitiligo cases and controls. Unconditional univariate analyses and multivariate logistic regression analyses were performed to obtain the crude and adjusted odds ratios (ORs) for the risk of vitiligo as well as the 95% confidence intervals (CIs). The multivariate adjustment included age and gender. The D′ value for linkage disequilibrium between the three *iNOS* polymorphisms and the haplotypes frequencies were calculated by SHEsis (http://analysis.bio-x.cn/myAnalysis.php) [24]. The genotype data for each polymorphism was further stratified by subgroups of sex, stage, type, onset age, family history, and with/without other autoimmune diseases. The interaction between the three *iNOS* polymorphisms was evaluated by using multivariate logistic regression models. Statistical significance was established at a *P* value of 0.05. Two-side tests of statistical significance were performed by using the SAS software (version 9.1; SAS Institute, Inc., Cary, North Carolina).

## Results

### Characteristics of the Study Population

The frequency distributions of selected characteristics of the controls and vitiligo patients are summarized in table 1 (Table 1). The age of the patients ranged from 2 to 95 years (24.9±13.9, mean±SD) and the controls are from 1 to 75 years (26.2±13.7) (*P* = 0.703). The frequency distributions in men and women were 55.3 and 44.7%, respectively in the cases, and 54.1 and 45.9%, respectively in the controls (*P* = 0.675). Among the cases were 43 (5.8%) segmental vitiligo and 706 (94.2%) nonsegmental vitiligo patients and 623 (83.2%) active and 126 (16.8%) stable vitiligo patients. One hundred and twenty eight (17%) vitiligo patients were considered to have a family history and 28 (4.2%) cases have other auto-immune diseases. Patients were considered to have early onset vitiligo (n = 384, 61.4%) if the age of onset was prior to 20 years old [25].

**Table 1:** Clinical characteristics of the 749 Chinese vitiligo patients and 763 healthy controls.

	Vitiligo(N/%) (N = 749)	Healthy controls (n/%) (n = 763)
Average age (year, mean±S.D.)	24.9±13.9	26.2±13.7
Number of female/male	335(0.45)/414(0.55)	350(0.46)/413(0.54)
Early onset/late onset	462(0.62)/287(0.38)	
Active/stable	623(0.83)/126(0.17)	
Nonsegmental/segmental	706(0.94)/43(0.06)	
With/without family history	128(0.17)/621(0.83)	
With/without other autoimmune diseases	28(0.04)/721(0.96)	

### Association between *iNOS* Genotypes and the Risk of Vitiligo

As shown in Table 2, the distribution of all genotypes among controls was compatible with the Hardy-Weinberg equilibrium (χ^2^: *P* = 0.877 for *iNOS*-1173 *C*→*T*; *P* = 0.133 for *iNOS*-954 *G*→*C*; *P* = 0.080 for *iNOSEx*16+14 *C*→*T*). The differences in *iNOS*-1173 and *Ex*16+14 genotyping distributions between vitiligo cases and controls were not statistically significant (*P* = 0.542 and *P* = 0.736, respectively). Similarly, the *iNOS*-1173 *T* allele and *Ex*16+14 *T* allele did not differ significantly between cases and controls (12.6% *vs.*11.4%, *P* = 0.329 and 13.1% *vs.*15.5%, *P* = 0.068, respectively). However, the *iNOS*-954 variant *C* allele frequency was significantly higher among cases than among controls (8.9% *vs.*6.7%, *P* = 0.028), and the frequency of the *iNOS*-954 (*GC*+*CC*) combined genotype was also significantly higher in cases than in controls (16.6% *vs.*12.6%, *P* = 0.029).

**Table 2:** Genotypic frequencies of *iNOS* polymorphisms in cases and controls and their associations with risk of vitiligo.

Genotypes	Cases (N = 749)		Controls (n = 763)^a^		adjusted OR (95% CI)^b^	*P* c
	N	%	n	%		
*iNOS-954*						
*GG*	625	83.4	667	87.4	1.00	0.087
*GC*	115	15.4	90	11.8	1.35(1.01–1.81)	
*CC*	9	1.2	6	0.8	1.49(0.52–4.23)	
*GC+CC*	124	16.6	96	12.6	1.36(1.02–1.81)	0.029
*C* allele		8.9		6.7		0.028
*iNOS-1173*						
*CC*	574	76.6	599	78.5	1.00	0.542
*CT*	161	21.5	154	20.2	1.09(0.85–1.40)	
*TT*	14	1.9	10	1.3	1.42(0.62–3.23)	
*CT+TT*	597	79.7	637	83.5	1.11(0.87–1.41)	0.058
*T* allele		12.6		11.4		0.329
*iNOSEx16+14*						
*CC*	571	76.2	569	74.6	1.00	0.736
*CT*	160	21.4	173	22.7	0.92(0.72–1.18)	
*TT*	18	2.4	21	2.8	0.84(0.44–1.60)	
*CT+TT*	178	23.7	194	25.4	0.91(0.72–1.16)	0.453
*T* allele		13.1		15.5		0.068

CI, confidence interval; OR, odds ratio.

a The observed genotype frequencies among the controls were in agreement with the Hardy–Weinberg equilibrium (χ^2^: *P* = 0.977 for *iNOS*-1173 *C*→*T*; *P* = 0.133 for *iNOS*-954 *G*→*C*; *P* = 0.080 for *iNOSEx*16+14 *C*→*T*).

b ORs were obtained from a multivariate logistic regression model with adjustment for age and sex.

c Adjustment for age and sex.

When we used the *iNOS*-954 *GG* genotype as the reference group, we found a statistically significant increased risk of vitiligo was associated with the -954 combined (*GC*+*CC*) genotype (adjusted OR = 1.39; 95% CI = 1.11–1.74). However, when *iNOSEx*16+14 *CC* and *iNOS*-1173 *CC* were used as reference groups, no significant risk was associated with any other genotypes.

### Stratification analysis of the *iNOS* Polymorphisms and Known Risk Factors of Vitiligo

To investigate the effects of *iNOS* polymorphisms on vitiligo, we performed an additional stratification analysis with the dichotomized genotypes groups. We observed significant association with *iNOS*-954 combined (*GC*+*CC*) genotype in subgroups that were with active vitiligo (adjusted OR = 1.35; 95% CI = 1.01–1.82), that were diagnosed with nonsegmental vitiligo (adjusted OR = 1.36; 95% CI = 1.02–1.82) and that had no other autoimmune diseases (adjusted OR = 1.39; 95% CI = 1.04–1.85) (Table 3). We also performed the stratified analysis for *iNOS*-1173 and *Ex*16+14, but no statistically significant results were found (data not shown).

**Table 3:** Stratification analysis of the *iNOS* -954 genotypes and vitiligo risk by selected variables.

Variables	*iNOS*-954 (case/control)				Adjusted OR (95% CI)^a^	*P* b
	*GG*		*GC+CC*			
	N	%	n	%		
Total	625/667	48.4/51.6	124/96	56.4/43.6	1.36 (1.02–1.81)	0.029
Onset age(years)						
≤20	384/667	61.4/**-**	78/96	62.9/**-**	1.08 (0.76–1.53)	0.087
>20	241/667	38.6/**-**	46/96	37.1/**-**	1.47 (0.97–2.22)	0.146
Sex						
Male	345/362	55.2/54.3	69/51	55.6/53.1	1.39 (0.93–2.05)	0.078
Female	280/305	44.8/45.7	55/45	44.4/46.9	1.32 (0.86–2.02)	0.187
Stage						
Stable	105/667	16.8/**-**	21/96	16.9/**-**	1.32 (0.79–2.22)	0.209
Active	520/667	83.2/**-**	103/96	83.1/**-**	1.35 (1.00–1.82)	0.037
Type						
Nonsegmental	589/667	94.2/**-**	117/96	94.4/**-**	1.36 (1.02–1.82)	0.030
Segmental	36/667	5.8/**-**	7/96	5.6/**-**	1.09 (0.46–2.55)	0.480
Family history						
Yes	105/667	16.8/**-**	23/96	18.5/**-**	1.42 (0.86–2.35)	0.097
No	520/667	83.2/**-**	101/96	81.5/**-**	1.33 (0.98–1.80)	0.051
Autoimmune diseases						
With	26/667	4.2/**-**	2/96	1.6/**-**	0.57 (0.13–2.44)	0.391
Without	599/667	95.8/**-**	122/96	98.4/**-**	1.39 (1.04–1.85)	0.018

a Odds ratios (ORs) were obtained from a multivariate logistic regression model with adjustment for age and sex. 95% CI, 95% confidence interval.

b Adjustment for age and sex.

### Association between *iNOS* Haplotypes and the Risk of Vitiligo

The Linkage Disequilibrium tests showed that the three polymorphisms of *iNOS* gene were in Linkage Disequilibrium (D′ = 0.897, *P*<0.01 for *iNOS*-1173 and -954; D′ = 0.613, *P*<0.01 for *iNOS*-1173 and *Ex*16+14; D′ = 0.392, *P*<0.01 for *iNOS* -954 and *Ex*16+14). There were eight possible *iNOS* haplotypes derived from the known genotypes, whereas those frequency<0.03 were ignored in analysis (i.e. *C*_-1173_ *C*_-954_ *T_Ex_*_16+14_, *T*_-1173_ *G*_-954_ *T_Ex_*_16+14_, *T*_-1173_ *C*_-954_ *T_Ex_*_16+14_, *T*_-1173_ *C*_-954_ *C_Ex_*_16+14_). As shown in Table 4, the *iNOS C*_-1173_ *C*_-954_ *C_Ex_*_16+14_ haplotype frequency was significantly different between vitiligo cases and controls (7.3% *vs.*5.3%, respectively; *P* = 0.016). Moreover, the haplotype *C*_-1173_ *C*_-954_ *C_Ex_*_16+14_ was associated with a significantly increased risk of vitiligo (adjusted OR = 1.44, 95% CI = 1.01–1.74).

**Table 4:** Frequencies of the *iNOS* haplotypes among the cases and controls and their associations with risk of vitiligo.

*iNOS* Haplotype	Case(%)^a^	Control(%)^a^	χ^2^	*P*	OR(95%CI)
*C_-1173_G_-954_C_Ex16+14_*	1021(68.1)	1060(69.4)	0.083	0.772	0.98 (0.83–1.14)
*C_-1173_G_-954_T_Ex16+14_*	172(11.5)	200(13.1)	1.455	0.228	0.88 (0.70–1.09)
*T_-1173_G_-954_C_Ex16+14_*	155(10.3)	163(10.7)	0.032	0.859	0.98 (0.78–1.24)
*C_-1173_C_-954_C_Ex16+14_*	109(7.3)	80(5.3)	5.753	0.016	1.44 (1.01–1.74)

a Frequency<0.03 (i.e., *C*_-1173_ *C*_-954_ *T_Ex_*_16+14_, *T*_-1173_ *G*_-954_ *T_Ex_*_16+14_, *T*_-1173_ *C*_-954_ *T_Ex_*_16+14_, *T*_-1173_ *C*_-954_ *C_Ex_*_16+14_) in both control & case has been ignored in analysis.

### The Interaction among the *iNOS* Polymorphisms and Association between *iNOS* Combined Genotypes and Risk of Vitiligo

Considering the potential joint effects of the three polymorphisms of *iNOS*, we further evaluated the interaction among the *iNOS* polymorphisms and the association between the combined genotypes of *iNOS*-1173, -954, and *Ex*16+14 and the risk of vitiligo, with the multivariate logistic regression models. Firstly, we analyzed any two of the three polymorphisms. We found the interaction between *iNOS*-1173, -954, and *Ex*16+14 was not statistically significant (*P* = 0.311, *P* = 0.150, *P* = 0.202, respectively). We next analyzed the combined genotypes of the three *iNOS* polymorphisms, but we found no statistically significant relationship between any two of the three polymorphisms or among the three iNOS polymorphisms (data not shown).

### Association between Serum iNOS Activity and the Risk of Vitiligo

**Figure 1:**
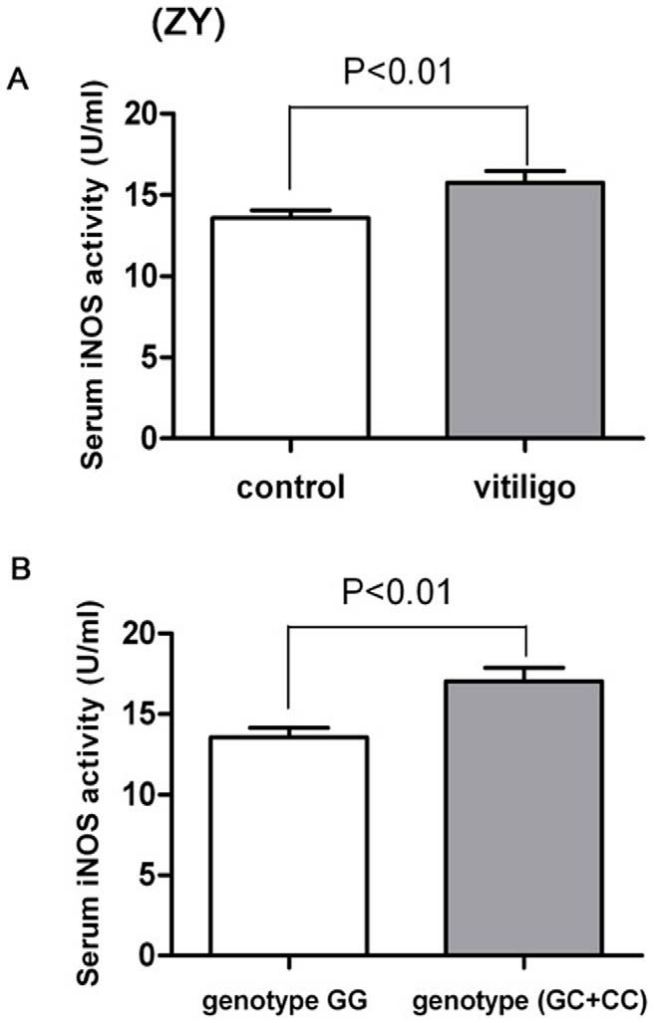
Serum iNOS activity and correlations to vitiligo genetype. (A) The serum iNOS activity in vitiligo patients' group is significantly higher than that in the normal control group (*P*<0.01). (B) Compared with the *iNOS*–954 protective genotype *GG* group, the risk genotype (*GC*+*CC*) group has the higher serum iNOS activity (*P*<0.01).

In order to analysis the serum iNOS activity and risk of vitiligo, we compared the serum iNOS activity in 89 vitiligo patients with the 89 normal control samples. We found the serum iNOS activity in vitiligo patients' group is significantly higher than that in the normal control group (15.40±3.98 U/ml *vs.*13.29±2.45 U/ml; *u* = 6.58, *P*<0.01) (Figure 1A). Furthermore, to examine the relationship between the *iNOS*-954 gene polymorphism and the serum iNOS activity in vitiligo patients, we also studied the serum iNOS activity in differential genotype of *iNOS*-954. Because the number of risk genotype *CC* group is very small (3 samples), we only examined the serum iNOS activity in *iNOS*-954 protective genotype *GG* group with the risk genotype (*GC*+*CC*) group. Our results showed that compared with the *iNOS*-954 protective genotype *GG* group, the risk genotype (*GC*+*CC*) group has the higher serum iNOS activity (16.87±4.20 U/ml *vs.*13.83±3.56 U/ml; *t* = 7.48, *P*<0.01) (Figure 1B).

### Logistic Regression Analysis of iNOS Activity in Vitiligo Patients and Controls

As shown in Table 5, we performed a logistic regression analysis of serum iNOS activity in vitiligo patients and controls. When we dichotomized the iNOS activity by the median activity of the controls, we found an increased risk for vitiligo was associated with the higher activity (adjusted OR = 2.14, 95% CI = 1.88–2.45). According to the iNOS activity of controls, we further divided the iNOS activity into three decile. When we used efficient (lower tertile) activity as the reference, the suboptimal (upper tertile) activity was associated with an increased risk of vitiligo (adjusted OR = 1.64, 95% CI = 1.35–1.86; *P*_trend_<0.001).

**Table 5:** Logistic regression analysis of iNOS activity in vitiligo patients and controls.

Activity	Cases(N = 89)		Controls(n = 89)		adjusted OR^a^ (95% CI)
	N	%	n	%	
By median					
<14.05	28	31.4	48	53.9	1.00
≥14.05	61	68.6	41	46.1	2.14(1.88–2.45)
By tertile					
≤13.17	15	16.9	32	36.0	1.00
13.17–14.83	33	37.1	35	39.3	1.14(0.88–1.36)
≥14.83	41	46.0	22	24.7	1.64(1.35–1.86)
Trend test	-	-	-	-	*P* b<0.001

a Odds ratios (ORs) were obtained from a logistic regression model with adjustment for age and sex; 95% CI, 95% confidence interval.

b Adjusted for age and sex.

### Risk of Vitiligo Associated with *iNOS*-954 *G*→*C* Genotypes by iNOS Activity

Furthermore, we estimated the risk of vitiligo associated with *iNOS*-954 genotypes by iNOS activity (Table 6). We divided *iNOS*-954 *G*→*C* genotypes into two categories, 0 risk genotype (-954 *GG*) and 1–2 risk genotypes (-954 *GC* or -954 *CC*). When the -954 *GG* was used as the reference, the individuals with 1–2 risk genotypes (-954 *GC* or -954 *CC*) and higher iNOS activity (≥14.05) showed more increased risk of vitiligo (adjusted OR = 4.53, 95% CI = 2.71–7.44). When we divided the iNOS activity into three decile according to the controls' activity, the -954 *GC* or *CC* genotype combined higher iNOS activity was associated with increased risk of vitiligo (≥14.83, adjusted OR = 2.40, 95% CI = 1.35–4.03).

**Table 6:** Risk of vitiligo associated with *iNOS*-954 *G*→*C* genotypes by iNOS activity.

Activity	*iNOS*-954 *G*→*C* (case/control)			
	0 risk genotype	OR(95% CI)^a^	1–2 risk genotype	OR(95% CI)^a^
By median				
<14.05	18/50	1.00	6/12	0.81(0.25–2.68)
≥14.05	45/22	1.17(0.33–1.62)	20/5	4.53(2.71–7.44)
By tertile				
≤13.17	11/38	1.00	2/6	0.41(0.14–2.76)
13.17–14.83	19/22	1.02(0.84–1.32)	10/5	1.07(0.74–1.80)
≥14.83	33/12	1.61(0.21–2.06)	14/4	2.40(1.35–4.03)
Trend test	-	-	-	*P* b<0.001

a Odds ratios (ORs) were obtained from a logistic regression model with adjustment for age and sex; 95% CI, 95% confidence interval.

b Adjusted for age and sex.

## Discussion

In this hospital-based case-control study, we investigated the associations of the *iNOS*-1173 *C*→*T*, -954 *G*→*C*, and *Ex*16+14*C*→*T* polymorphisms with risk of vitiligo in Han Chinese populations. We found that compared to the *iNOS*-954 *GG* genotype, the *iNOS*-954 combined (*GC*+*CC*) genotypes were associated with an increased risk of vitiligo. The association was more pronounced in patients with active vitiligo, nonsegmental vitiligo and vitiligo without other autoimmune disorders. This suggests that *iNOS*-954 gene polymorphism may have a greater effect on these subgroups. The differences maybe due to the reduction in the number of observations in some strata or may reflect true susceptible subgroups. Further exploration is needed to determine whether the effects found here are an aberration, and what the exact mechanisms underlying such effects are.

Compared with the previously published data, the *iNOSEx*16+14 *T* allele frequencies were similar to the frequencies in other Chinese controls and Japanese [26], [27]. However, we found the *iNOS*-954 *C* allele frequency was lower in Han Chinese than in African [22], which indicated that the genotype distributions of the *iNOS*-954 polymorphism vary with ethnicity.

The *iNOS*-954 polymorphism is located in the *iNOS* promoter region, which could be an activator of gene expression [22]. *INOS*-954 *C* allele, but not the *iNOS*-954 *G* allele, may have a higher affinity to a DNA binding protein, thus, transcriptional activity and mRNA levels were increased in the *iNOS*-954 *C* allele. Then the NOS activity and NO production were increased. Our result certified *iNOS*-954 *C* allele can increase iNOS activity.

To the best of our knowledge, this is the first study on the association between the *iNOS* polymorphisms and vitiligo in the Han Chinese population. An early event in the onset of vitiligo appears to involve the overproduction of tetrahydrobiopterin, which leads to the accumulation of a potent inhibitor of melanin biosynthesis [28]. The synthesis of tetrahydrobiopterin is cytokine induced and it is an essential cofactor in the enzymatic activity of iNOS [29]. LPS/cytokines can stimulate normal human melanocytes express iNOS. This enzyme might therefore be involved in the altered melanin production associated with post-inflammatory hypopigmentation [30]. In vitiligo, the increase of iNOS activity caused by overexpression of the tetrahydrobiopterin or LPS/cytokines can produce plenty of NO generation. NO has been reported to contribute to the loss of melanocytes in vitiligo by reducing *de novo* attachment of melanocytes to the extracellular matrix components. Moreover, increased iNOS activity induces NO production and O_2_^−^, which result in the accumulation of hydrogen peroxide. High of hydrogen peroxide can lead to melanocytes destruct and depigmentation ultimately [31]. The increased NOS activity had been confirmed in vitiligo affected/nonaffected melanocytes and keranocytes. Our study also confirmed that the increased iNOS activity was related with the onset of vitiligo indirectly. However, the specific mechanisms of iNOS involved in the pathogenesis of vitiligo still need further research.

In summary, we provide evidence that *iNOS* polymorphisms may influence the risk and clinical progression of vitiligo in Han Chinese populations. A statistically significantly increased risk of vitiligo was associated with the *iNOS*-954 (*GC*+*CC*) genotype compared with the -954 *GG* genotype, which was more pronounced among vitiligo patients with the following characteristics: non-segmental, active vitiligo and without other autoimmune diseases. But no evident risk was associated with the *iNOS*-1173 and *Ex*16+14 polymorphisms. Furthermore, we found the serum iNOS activity was significantly higher in vitiligo and was increased in *iNOS*-954 combined genotype (*GC*+*CC*) compared with -954 *GG* genotype. Nevertheless, better-designed and larger prospective studies are needed to confirm these findings, and more detailed environmental exposure data are necessary to further test potential gene-environment interactions.
